# Selenomethionine alleviates aortic dissection via PGC-1α/NRF2/TFAM-mediated mitochondrial biosynthesis against ferroptosis: an experimental study

**DOI:** 10.1097/JS9.0000000000003561

**Published:** 2025-11-10

**Authors:** Shengwei Lai, Handai Qin, Xinhao Wang, Yiming Bi, Zhiwei Lai, Shuaifei Ji, Wei Guo

**Affiliations:** a Department of Vascular Surgery, First Medical Centre of Chinese PLA General Hospital, Beijing, China; b Graduate School, Chinese PLA General Hospital, Beijing, China; c School of Ocean Energy, Tianjin University of Technology, Tianjin, China; d Department of Burn and Plastic Surgery, PLA Rocket Force Characteristic Medical Center, Beijing, China

**Keywords:** aortic dissection, ferroptosis, mitochondrial biosynthesis, selenomethionine, vascular smooth muscle cells

## Abstract

**Background:**

Aortic dissection (AD) is a cardiovascular disease with an extremely high mortality rate, and there is currently a lack of specific therapeutic drugs clinically. Selenomethionine (Set), known for its antioxidant and anti-inflammatory properties, has demonstrated promising potential in the treatment of cardiovascular diseases. Here, its therapeutic efficacy in AD and underlying mechanisms were explored.

**Methods:**

The mouse model was established by β-aminopropionitrile fumarate induction. The effects of Set on aortic dilation, histopathology, and survival rate were evaluated. *In vitro*, platelet-derived growth factor-BB (PDGF-BB)-induced phenotypic switching of vascular smooth muscle cells (VSMCs) was utilized. EdU assay, Transwell migration assay, and lipid peroxidation experiments were conducted to analyze the impact of Set. Key molecular mechanisms were further validated through single-cell sequencing, transcriptomic analysis, and small interfering RNAs knockdown techniques.

**Results:**

*In vivo* experiments demonstrated that Set significantly reduced the incidence and mortality of AD, suppressed aortic dilation, alleviated elastic fiber fragmentation, and inhibited collagen hyperplasia. *In vitro*, Set effectively attenuated PDGF-BB-induced proliferation, migration, and phenotypic switching of VSMCs. Single-cell sequencing revealed marked enrichment of ferroptosis-related genes in VSMCs from patients with AD. Transcriptomic analysis indicated that Set substantially inhibited ferroptosis signaling and activated mitochondrial biogenesis pathways. Mechanistically, Set activated the PGC-1α/NRF2 pathway, upregulated GPX4 expression, reduced lipid peroxidation, and improved mitochondrial function, ultimately suppressing phenotypic switching of VSMCs. Notably, knockdown of GPX4 or PGC-1α weakened the inhibitory effects of Set on VSMCs’ phenotypic switching.

**Conclusions:**

Set alleviated AD progression by suppressing ferroptosis-mediated phenotypic switching of VSMCs through targeted activation of the PGC-1α/NRF2/TFAM axis.

**HIGHLIGHTS**

- Aortic dissection (AD) is a cardiovascular disease with an extremely high mortality rate, and there is currently a lack of specific therapeutic drugs clinically.
- Selenomethionine (Set), known for its antioxidant and anti-inflammatory properties, has shown promising potential in the treatment of AD.
- Set alleviated AD progression by suppressing ferroptosis-mediated phenotypic switching of vascular smooth muscle cells.
- Set inhibited ferroptosis by regulating the PGC/NRF/TFAM-mediated mitochondrial biosynthesis pathway.

## Introduction

Aortic dissection (AD) is a highly lethal cardiovascular disease, with 3–4 cases per 100 000 people annually^[1]^. The most common treatments for AD are endovascular repair and open surgery. Data indicate that the 48 h mortality rate of patients with AD after emergency surgery is 4.4%, and the in-hospital mortality rate related to emergency surgery is as high as 19.7%^[2,3]^. However, there is no specific effective drug for AD in clinical practice. Currently, the drug treatment for AD focuses on controlling blood pressure (such as beta-blockers combined with sodium nitroprusside), reducing myocardial contractility and analgesia. Therefore, there is an urgent clinical need to discover new therapeutic targets and drugs for AD. Recent animal experiments have demonstrated that some drugs can significantly inhibit the occurrence and development of AD, such as melatonin, colchicine, Senkyunolide I, and cannabidiol^[4–7]^.

Pathologically, AD is associated with degenerative changes in the vascular media layer. Key features include the phenotypic transformation of vascular smooth muscle cells (VSMCs), as well as extracellular matrix alterations, such as the disruption of the elastic fiber layer, increased collagen fibers, and inflammatory cell infiltration. Multiple studies have reported that inhibiting the phenotypic switching of contractile VSMCs (the normal phenotype) can significantly reverse AD progression^[8–11]^.

Ferroptosis is a newly discovered form of cell death primarily triggered by iron overload^[12–15]^. Excessive intracellular iron ions catalyze lipid peroxidation via the Fenton reaction, resulting in the accumulation of toxic lipid peroxides^[16]^. Under normal circumstances, glutathione peroxidase 4 (GPX4) can prevent their accumulation. However, when GPX4 activity is inhibited or glutathione availability is insufficient, these toxic lipid peroxides accumulate excessively, damaging cell membranes and ultimately inducing cell death^[17]^. This mode of cell death plays a critical role in the pathogenesis of cardiovascular diseases and offers novel therapeutic targets for related disorders^[18,19]^. In patients with AD, altered expression of ferroptosis-associated genes has been identified^[20]^. It has been reported that nicotine promotes the progression of AD by activating ferroptosis signaling^[21]^. Inhibiting ferroptosis signaling can also significantly ameliorate AD progression^[22,23]^.

Peroxisome proliferator-activated receptor gamma coactivator 1α (PGC-1α) and nuclear factor E2-related factor 2 (NRF2) play critical roles in the cellular antioxidant defense and mitochondrial biogenesis, exhibiting a close functional association with GPX4^[24]^. Upon activation, PGC-1α triggers a cascade of signaling pathways, including those regulating NRF2. As a master transcription factor for intracellular antioxidant responses, NRF2 translocates to the nucleus upon activation, binds to specific DNA motifs, and upregulates the expression of the downstream antioxidant enzyme GPX4^[25]^.

Selenomethionine (Set), a selenium-containing amino acid, is widely distributed in various selenium-rich foods and exhibits diverse biological activities, including antioxidant, anti-inflammatory, antiaging, and ferroptosis inhibition^[26,27]^. Additionally, Set can promote mitochondrial biogenesis by enhancing PGC-1α expression and mitochondrial function^[28]^. These multiple functions endow Set high therapeutic potential for the prevention and treatment of cardiovascular diseases. For example, Set supplementation can enhance vascular function and alleviate atherosclerosis. Nevertheless, the effect of Set on AD progression remains unclear and warrants further investigation.

In this study, we aimed to explore the therapeutic effects and molecular mechanisms of Set on AD. We discovered that Set reduced ferroptosis and enhanced mitochondrial biogenesis via activation of the PGC-1α/NRF2 signaling pathway. These effects further suppressed the phenotypic transformation of VSMCs and, ultimately, alleviated AD progression. Our research presents novel insights into AD treatment and provides a potential pharmacological strategy. This article adheres to the TITAN Guidelines 2025^[29]^.

## Material and methods

### Animal models and drug administration

Three-week-old specific pathogen-free male C57BL/6 mice (~10–15 g) were sourced from SiPeiFu Biotechnology Co., Ltd. in Beijing, China, and housed in standard cages under a pathogen-free environment with a 12-h light/dark cycle. Animal experiments adhered to the National Institutes of Health (NIH) Guide for the Care and Use of Laboratory Animals (8th edition). β-Aminopropionitrile fumarate (BAPN, Sigma-Aldrich) was used to induce the AD mouse model. Mice were sequentially numbered and randomly divided into four groups using a randomization table for a 4-week treatment: (1) Control group receiving intraperitoneal normal saline; (2) Control + Set (CAS No.1464-42-2, MCE) group receiving intraperitoneal Set (200 µg/kg, diluted with saline); (3) BAPN group receiving oral BAPN (0.4% in drinking water) and intraperitoneal normal saline; (4) BAPN + Set group receiving both oral BAPN and intraperitoneal Set. During the experiment, the mice’s body weights were measured every 3 days. AD mice were diagnosed based on the following criteria: tear of the aortic wall with intramural bleeding and false lumen formation, the discovery of thoracic thrombosis at autopsy, or local artery dilation greater than 50% of normal. Animal procedures were approved by the Chinese PLA General Hospital Ethics Committee. Additionally, animal studies complied with the ARRIVE guidelines^[30]^.

### Ultrasound and morphologic examination

The mice were anesthetized with 2% isoflurane on day 28 of intervention treatment, and aortic diameters were measured *in vivo* using the Vevo 2100 high-resolution ultrasound system (VisualSonics, Toronto, Canada). Mice were subsequently euthanized and perfused intracardially with 4% paraformaldehyde (PFA). The aortas, spanning from the aortic root to the iliac artery bifurcation, were surgically dissected to enable precise *in vitro* quantification of aortic diameters.

### Histology and immunofluorescence staining

Dissected mouse aortas underwent sequential processing: postfixation in 4% PFA solution for 48 h, followed by paraffin embedding, and finally sectioning into 5-μm-thick histological slices. Histopathological evaluation was performed using standard hematoxylin–eosin (H&E) staining protocols. Collagen content was quantified using Masson’s trichrome staining protocol (Beyotime Biotechnology, Shanghai, China). Elastic Verhoeff–van Gieson (EVG) staining was conducted to assess elastin integrity using resorcinol–fuchsin and methylene blue solution. Elastin degradation was graded on a 1–4 scale as previously described: grade 1 (intact structure), grade 2 (minor interruptions), grade 3 (moderate fragmentation), and grade 4 (severe rupture). Immunofluorescence staining was performed with antibodies against αSMA, GPX4, MDA, 4-HNE, PGC-1α, MMP2, and MMP9. Nuclei were counterstained with DAPI. Aortic sections were imaged using a Leica fluorescence microscope. ImageJ software (developed by the NIH) was used to quantify fluorescence intensity in each group.

### Cell culture

Human VSMCs were sourced from Procell Technology Co. (Cat. No. CP-H081). Cells were cultured in Dulbecco’s Modified Eagle Medium (Gibco) supplemented with 10% fetal bovine serum (Gibco) and 1% penicillin–streptomycin (Gibco), under standardized culture conditions (37ºC, 5% CO_2_, 95% humidity), with subculturing performed every 3–4 days to preserve optimal density.

### Western blot

Antibodies against the following targets were used: α-smooth muscle actin (αSMA, 1:1000, ab7817), osteopontin (OPN, 1:1000, ab283656), cyclin D1 (1:10 000, ab134175), vimentin (1:2000, ab92547), GPX4 (1:1000, ab231174), ACSL4 (1:10 000, ab155282), malondialdehyde (MDA, 1:1000, ab243066), 4-hydroxynonenal (4-HNE, 1:1000, ab46545), and mitochondrial transcription factor A (TFAM, 1:1000, ab272885) from Abcam; transgelin (SM22, 1:5000, 10 493-1-AP), PCNA (1:2000, 24 036-1-AP), PGC-1α (1:5000, 66 369-1-Ig), and NRF2 (1:1000, 80 593-1-RR) from Proteintech. Equivalent protein quantities were divided through 10% sodium dodecyl sulfate polyacrylamide gel electrophoresis (SDS-PAGE) and subsequently immobilized on polyvinylidene fluoride (PVDF) membranes. Membranes were incubated with 5% BSA/TBS-Tween 20 (room temperature, 1 h) to block nonspecific binding, followed by overnight incubation with primary antibodies at 4ºC. After washing, horseradish peroxidase (HRP)-conjugated secondary antibodies (1:50 000) were incubated with the membranes at room temperature for 2 h. Chemiluminescent reagents were used for detection.

### EdU assay

VSMCs were seeded into 24-well plates overnight before the EdU experiment. Then, cells were cocultured with 20 ng/mL PDGF-BB alone or in combination with 50 μM Set. Subsequently, cell proliferation was quantified through EdU staining assay kit (C0078S, Beyotime) as instructed by the supplier.

### CCK-8 assay

Cell viability was estimated by the Cell Counting Kit (CCK)-8 assay (C0037, Beyotime). Cells were plated in 96-well plates (~1 × 10^4^ cells per well in 100 µL medium). Following a 24-h cell culture period, PDGF-BB with or without Set treatment was added. Subsequently, CCK-8 solution (10 µL) was added to each well and incubated for 1 h in the dark. Subsequently, absorbance at 450 nm readings was measured using a microplate reader.

### Reverse transcription–quantitative polymerase chain reaction

RNA was extracted using Trizol reagent (R0016, Beyotime) and then reverse-transcribed into cDNA. Reverse transcription–quantitative polymerase chain reaction (RT-qPCR) was performed using SYBR Green qPCR Mix according to the manufacturer’s instructions. *GAPDH* served as the reference gene for normalization. RT-qPCR primer sequences are listed in Supplemental Digital Content Table S1, available at: http://links.lww.com/JS9/F489.

### Transwell migration assay

The 24-well plates with cell chambers of 8 µm pore size (FTW067-12Ins, BeyoGold) were used to detect cell migration. VSMCs (1 × 10^5^ cells per well) were seeded in the upper chamber of the Transwell system using serum-free medium. Subsequently, the lower chamber was filled with complete medium. Cells were incubated at 37°C, then fixed and stained with crystal violet. Imaging and analysis of membrane lower surface cells were performed post-upper layer cell removal. Cell migratory capacity was assessed by averaging the number of migrating cells across three randomly selected fields of view.

### Wound healing test

A 1000-μL pipette tip was used to create a scratch once the cells in six-well plates reached 95%–100% confluence. The scratches were imaged using a Leica inverted microscope at 0 and 48 h, respectively. Scratches’ width was analyzed using ImageJ software.

### Lipid peroxidation assay

VSMCs were cultured in 24-well plates and treated with different groups. Subsequently, the cells were stained with BODIPY 581/591 C11 staining working solution (S0043S, Beyotime) for ~20 min. The cells were then washed with phosphate-buffered saline. Finally, the cells were observed using a Leica fluorescence microscope.

### Mitochondrial assays

To evaluate the mitochondrial membrane potential, VSMCs were cultured in 24-well plates and treated with different groups. Subsequently, VSMCs were stained with JC-1 staining working solution (C2006, Beyotime) for 20 min. After rinsing with the ice-cold 1 × JC-1 staining buffer, the cells were imaged using a Leica fluorescence microscope. To detect the intracellular ATP level, cells were also incubated in 24-well plates and subjected to group treatments. Next, the cells were mixed with the ice-cold ATP detection working solution (S0026, Beyotime), and the luminescence intensity (relative luminescence units) was measured using a luminometer.

### Single-cell sequencing

Single-cell sequencing data in this study were obtained from the NCBI GEO database (GSE189795, containing nine samples comprising five AD groups and four control groups). R software was employed for cell filtering, normalization, highly variable gene discovery, clustering analyses, and dimensionality reduction. Gene expression patterns were visualized using scatter plots, bubble charts, and bar graphs.

### RNA sequencing

VSMCs were cultured in six-well culture plates and treated with group-specific interventions. The experiment consisted of two groups: one treated with 20 ng/mL PDGF-BB (PDGF-BB group) and the other with 20 ng/mL PDGF-BB plus 50 μM Set (PDGF-BB + Set group). Three biological replicates were included per group, totaling six samples. RNA extraction was performed with TRIzol reagent. RNA quality was assessed using a Nanodrop 2000 spectrophotometer (Thermo Fisher Scientific), with A260/A280 absorbance ratios consistently exhibiting ~2.0. RNA integrity was verified using an Agilent Bioanalyzer 2100, with RIN values >8.0. Subsequently, differential gene expression analysis was analyzed using DESeq2, followed by functional annotation of differentially expressed genes (DEGs) via Gene Ontology (GO) and Kyoto Encyclopedia of Genes and Genomes (KEGG) pathway enrichment. Finally, heatmaps were constructed to visualize gene expression patterns.

### Small interfering RNA and transfection

Thermo Fisher Scientific provided small interfering RNAs (siRNA) targeting GPX4 (si-GPX4, Cat. No. AM16708, assay ID 10942) and PGC-1α (si-PGC-1α, Cat. No. AM16708, assay ID 10942 and 108385, respectively). A negative control siRNA (si-Control) was also purchased from Thermo Fisher Scientific (Cat. No. AM4611). VSMCs were cultured in 12-well plates. After reaching 50–70% confluence, they were transfected with si-GPX4 or si-PGC-1α using Lipofectamine RNAiMAX (Thermo Fisher Scientific). Additionally, cells were transfected with si-Control that did not target GPX4 as the negative-control group. Subsequently, the cells were harvested for downstream analytical procedures.

### Statistical analysis

First, the normality of data distribution was evaluated. Analysis of variance with subsequent Bonferroni correction as a *post hoc t*-test was used to evaluate differences across multiple experimental groups. The chi-square test or Fisher’s exact test was used to compare the incidence of thoracic AD. The Kaplan–Meier survival curves were employed to analyze the survival status of mice. All results were verified by at least three independent experiments. All statistical analyses were performed using GraphPad Prism 8 software. Statistical significance was defined as a *P* value *P* value < 0.05.

## Results

### Set alleviates vascular damage and inhibited AD development

**Figure 1.**
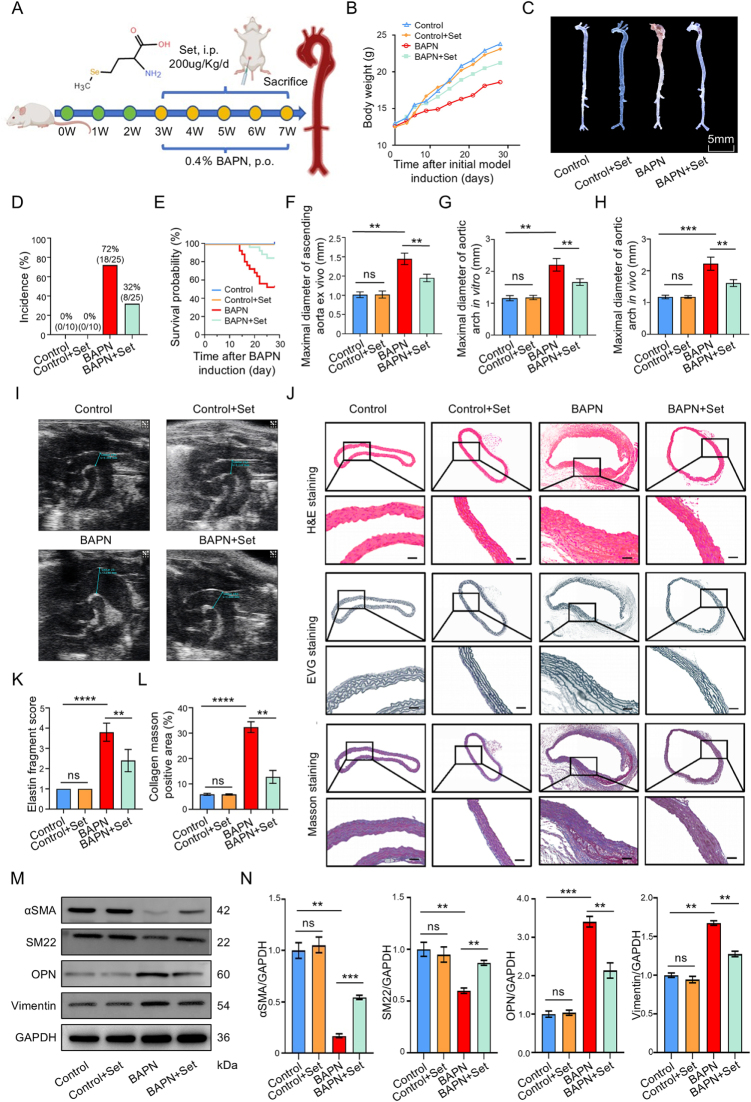
Set inhibited the progression of BAPN-induced AD mice. (A) Schematic diagram of the experimental protocol for Set treatment in AD mice. (B) Body weight changed over time in each group during the experimental period (*n* =10 in the Control and Control + Set groups, *n* = 25 in BAPN and BAPN + Set groups). (C) Representative photographs of aortic specimens from the Control, Control + Set, BAPN, and BAPN + Set groups. Scale bar = 5 mm. (D) The incidence of AD in the Control, Control + Set, BAPN, and BAPN + Set groups. (E) The survival rate in the Control, Control + Set, BAPN, and BAPN + Set groups. (**F**) Maximal diameter of ascending aorta *in vitro* in the Control, Control + Set, BAPN, and BAPN + Set groups (*n* = 5 per group). (**G**) Maximal diameter of aortic arch measured *in vitro* in the Control, Control + Set, BAPN, and BAPN + Set groups (*n* = 5 per group). (H) Maximal diameter of aortic arch measured using an ultrasonic system *in vivo* in the Control, Control + Set, BAPN, and BAPN + Set groups (*n* = 5 per group). (I) Typical ultrasound images of the aortic arch in the Control, Control + Set, BAPN, and BAPN + Set groups. (J) Representative images of H&E, EVG, and Masson staining in the Control, Control + Set, BAPN, and BAPN + Set groups. Scale bar = 50 μm. (K) Elastin fragment score in the Control, Control + Set, BAPN, and BAPN + Set groups (*n* = 5 per group). (L) Collagen Masson-positive area in the Control, Control + Set, BAPN, and BAPN + Set groups (*n* = 5 per group). (M) Representative images of western blots demonstrating differential protein expression of αSMA, SM22, OPN, vimentin, and GAPDH across the Control, Control + Set, BAPN, and BAPN + Set groups. (N) Quantified expression of αSMA, SM22, OPN, and vimentin from aortic specimens in the Control, Control + Set, BAPN, and BAPN + Set groups (*n* = 3 per group). *P < 0.05, **P < 0.01, ***P < 0.001, ****P < 0.0001; ns, not significant.

The experimental protocol for establishing the BAPN-induced AD models is schematically presented (Fig. 1A). Prior to formal experimentation, we conducted an exploratory dose-finding pilot study for Set via intraperitoneal administration, based on the documented safe dosage range in the literature. Experimental results demonstrated that, compared with high-dose group (500 μg/kg) and low-dose group (50 μg/kg), the medium-dose group (200 μg/kg) exhibited significantly greater inhibitory effects on AD pathological progression (Supplemental Digital Content Figure S1A, available at: http://links.lww.com/JS9/F489), and reduced AD incidence as well as mortality (Supplemental Digital Content Figure S1B and C, available at: http://links.lww.com/JS9/F489). Therefore, 200 μg/kg was selected as the therapeutic regimen for subsequent *in vivo* experiments. Longitudinal body weight monitoring was conducted throughout the 4-week experimental period, and results showed that BAPN intervention led to significant weight loss in AD model animals compared with the control group, but Set treatment significantly attenuated this weight loss (Fig. 1B). Aortic specimens were collected post-euthanasia for analysis, and significant dilatation and dissection were observed in the BAPN-induced AD models, while Set treatment could attenuate that vascular damage (Fig. 1C). BAPN group exhibited an AD incidence of 72.0% (18/25) with a mortality rate of 48.0%, whereas Set treatment effectively reduced both incidence (32.0%) and mortality rate (16.0%) of AD in BAPN-treated animals (Fig. 1D and E). Morphometric evaluations demonstrated significant aortic dilation in BAPN-treated mice, particularly in the ascending aorta and aortic arch regions, while Set treatment reversed these BAPN-induced pathological changes (Fig. 1F–I). Histopathological analysis revealed characteristic BAPN-induced aortic remodeling, including elastic fiber fragmentation and collagen deposition. Set treatment notably ameliorated these structural abnormalities, as shown in representative H&E, EVG, and Masson staining images (Fig. 1J–L). The phenotypic transformation of VSMCs plays a pivotal role in driving vascular dysfunction under pathological conditions. Key markers of the contractile phenotype in VSMCs include αSMA and SM22α, and the distinctive markers of synthetic phenotype include OPN and vimentin. These proteins are essential contributors to the contractile and synthetic characteristics of VSMCs. Western blot analysis further demonstrated that Set treatment reversed BAPN-induced abnormal phenotypic transformation, as evidenced by differential expression patterns of contractile and synthetic phenotypic markers (Fig. 1M and N).

### Set suppresses the proliferation, migration, and phenotypic switching of VSMCs

**Figure 2.**
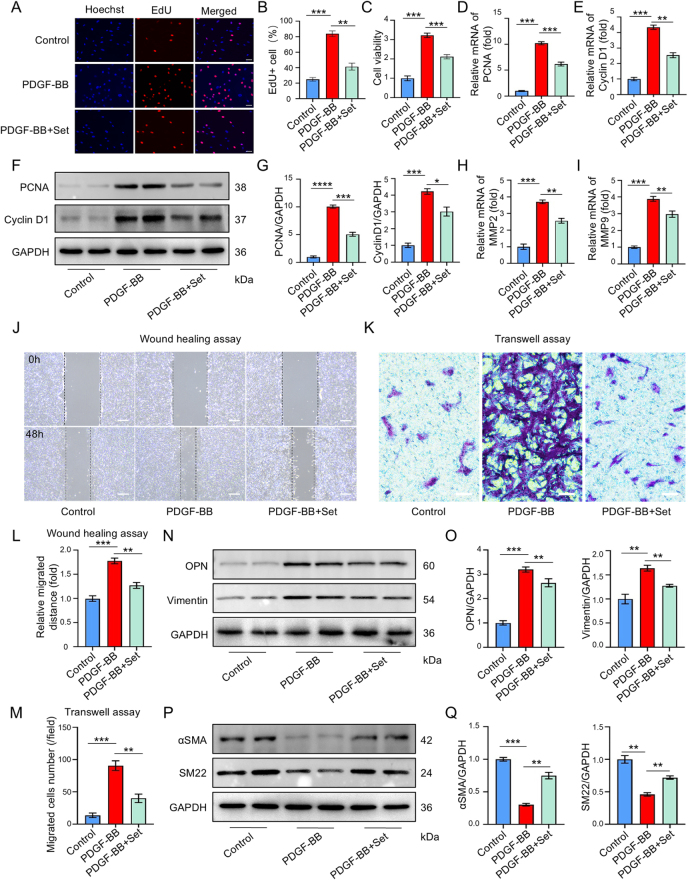
Set inhibited the proliferation, migration, and phenotypic transformation of VSMCs. (A) Representative images of fluorescent EdU assay in VSMCs from the Control, PDGF-BB, and PDGF-BB + Set groups. Hoechst was used to stain nuclei (blue). EdU dye was incorporated into proliferative cells (red). (B) EdU + cell count from the Control, PDGF-BB, and PDGF-BB + Set groups. (C) MTT assay in VSMCs from the Control, PDGF-BB, and PDGF-BB + Set groups. (D and E) RT-qPCR analyses of proliferative markers PCNA and cyclin D1 in VSMCs from the Control, PDGF-BB, and PDGF-BB + Set groups. (F) Representative western blots of Pcna, cyclin D1, and GAPDH in VSMCs from the Control, PDGF-BB, and PDGF-BB + Set groups. (**G)** Quantified protein analysis of PCNA and cyclin D1 in VSMCs from the Control, PDGF-BB, and PDGF-BB + Set groups. (H and I) RT-qPCR analyses of proliferative markers MMP2 and MMP9 in VSMCs from the Control, PDGF-BB, and PDGF-BB + Set groups. (J and L) Representative images of wound healing assay in the Control, PDGF-BB, and PDGF-BB + Set groups. The relative migrated distance at two time points (0 and 48 h) was measured and analyzed. (K and M) Representative images of Transwell assay from the Control, PDGF-BB, and PDGF-BB + Set groups. Migrated cells number was counted and analyzed. (N) Representative images of western blots of OPN, vimentin, and GAPDH in VSMCs from the Control, PDGF-BB, and PDGF-BB + Set groups. (O) Quantified protein analysis of OPN and vimentin in the Control, PDGF-BB, and PDGF-BB + Set groups. (P) Representative images of western blots of αSMA, SM22, and GAPDH in VSMCs from the Control, PDGF-BB, and PDGF-BB + Set groups. (Q) Quantified protein analysis of αSMA and SM22 in the Control, PDGF-BB, and PDGF-BB + Set groups. All biological experiments were repeated three times. The data were presented as the mean ± SD. * *P* < 0.05, ** *P* < 0.01, *** *P* < 0.001, **** *P* < 0.0001; ns, not significant.

*In vivo* studies have demonstrated that Set exerts effects on both contractile and synthetic markers. We hypothesized that the therapeutic impact of Set on AD may be associated with the functional changes of VSMCs as well as phenotypic transformation. Therefore, to investigate the effect of Set on abnormal VSMCs proliferation, migration, and phenotypic switching, and further explore the protective mechanism, the dedifferentiated VSMCs were induced through PDGF-BB treatment. Using CCK-8 assays, we systematically evaluated the dose-dependent effects of Set (0, 10, 25, 50, 100, 200 μM) on PDGF-BB-stimulated VSMCs proliferation. The results demonstrated progressive inhibition of cellular proliferation with increasing Set concentrations upto 200 μM, but low doses of Set have no significant influence (Supplemental Digital Content Figure S1D, available at: http://links.lww.com/JS9/F489). Notably, 50 μM Set effectively suppressed pathological proliferation while maintaining cell viability, establishing this concentration as optimal for subsequent experimental investigations. EdU incorporation assays also showed Set inhibited the enhanced VSMC proliferation induced by PDGF-BB (Fig. 2A and B). Cell viability analysis further supported these results (Fig. 2C). RT-qPCR results indicated that mRNA expression of proliferation markers PCNA and cyclin D1 in PDGF-BB-treated VSMCs was significantly upregulated, whereas Set treatment inhibited expression of these two markers (Fig. 2D and E). These results were subsequently confirmed by western blot analysis of protein (Fig. 2F and G). Set treatment also reversed dedifferentiation-induced upregulation of MMP2 and MMP9 (Fig. 2H and I), with concomitant inhibition of cell migration in wound healing (Fig. 2J and L) and Transwell assays (Fig. 2K and M). Western blot analysis demonstrated that Set concurrently suppressed expression of synthetic markers (OPN and vimentin) (Fig. 2N and O) while restoring expression of contractile markers (αSMA and SM22) in dedifferentiated VSMCs (Fig. 2P and Q). These findings collectively demonstrated the inhibitory effects of Set on abnormal VSMCs proliferation, migration, and phenotypic transition.

### Single-cell sequencing and transcriptomic analysis identified the potential signaling pathways involved in the therapeutic effect of Set on AD

**Figure 3.**
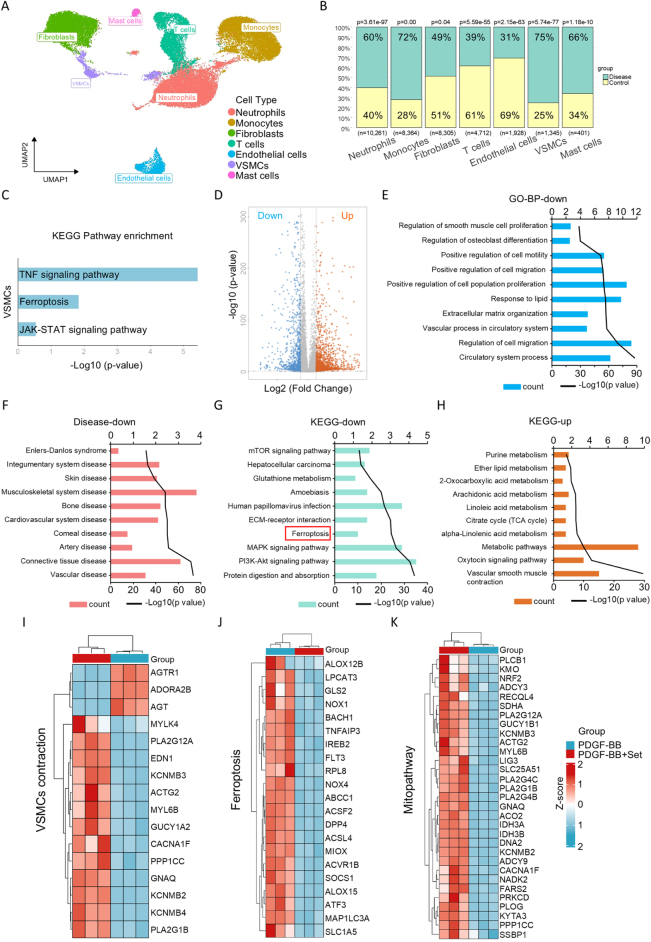
Single-cell and transcriptomic analysis reveal the molecular mechanism of Set in AD treatment. (A) UMAP dimensionality reduction identified eight distinct cell subpopulations in aortic tissues from patients with AD and healthy individuals, which were classified into seven major cell types: neutrophils, monocytes, fibroblasts, T cells, endothelial cells, VSMCs, and mast cells. (B) Single-cell proportion bar chart showing the relative abundance of cell subpopulations in patients with AD versus healthy individuals. (C) KEGG pathway analysis revealed that DEGs were enriched in TNF signaling, ferroptosis, and JAK-STAT pathways. (D) Volcano plots indicated the key DEGs in the comparison of PDGF-BB + Set-treated groups with PDGF-BB-treated groups. (E) GO analyses demonstrated that downregulated DEGs were mainly associated with VSMCs’ proliferation, migration, and lipid response. (F) Disease association analysis showed that downregulated DEGs were significantly enriched in vascular diseases, artery diseases, and cardiovascular system diseases. (G) KEGG enrichment analysis of downregulated DEG after Set treatment. (H) KEGG enrichment analysis of upregulated DEG after Set treatment. (I–K) Heatmap analysis of DEG in vascular smooth muscle contraction, ferroptosis, and mitopathway.

To comprehensively characterize the molecular landscape of cellular diversity and pathological processes in vascular abnormalities associated with AD, we conducted single-cell analysis of aortic specimens from both patients with AD and healthy controls. Using UMAP dimensionality reduction, we identified eight distinct subpopulations with significant differential expression patterns (Supplemental Digital Content Figure S2A, available at: http://links.lww.com/JS9/F489). Based on established cell type markers, these subpopulations were classified into seven major cell categories, including neutrophils, monocytes, fibroblasts, T cells, endothelial cells, VSMCs, and mast cells (Fig. 3A). A bubble plot was generated to visualize the expression profiles of typical markers for each cell type (Supplemental Digital Content Figure S2B, available at: http://links.lww.com/JS9/F489). Notably, aortic tissues from patients with AD exhibited a marked increase in the proportion of VSMCs, reaching 75% (Fig. 3B), indicating that VSMCs might play a pivotal role in the pathogenesis of AD. KEGG pathway analysis revealed that DEGs were predominantly enriched in TNF signaling pathway, ferroptosis, and JAK-STAT signaling pathway (Fig. 3C). These results suggested the potential pathophysiological mechanism of VSMCs in AD, and meanwhile, these signaling pathways may also mediate the effect of Set on AD development and VSMC function as well as phenotypic switching. To reveal the protective mechanism of Set, transcriptome sequencing of dedifferentiated VSMCs with or without Set treatment were performed. The volcano plot was generated to identify key DEG (Fig. 3D). GO functional enrichment analysis of biological processes demonstrated that downregulated genes were mainly enriched in VSMCs proliferation and migration (Fig. 3E). Notably, these downregulated genes were significantly enriched in vascular diseases, artery diseases, and cardiovascular system diseases (Fig. 3F). KEGG enrichment analysis further revealed that downregulated genes primarily enriched in PI3K/Akt signaling pathways, ferroptosis, and MAPK signaling pathways (Fig. 3G), consistent with our previous observation of ferroptosis activation in AD patient-derived VSMCs (Fig. 3C). This suggested that ferroptosis pathways seemed to be associated with the effect of Set on dedifferentiated VSMCs. In addition, upregulated genes were enriched in vascular smooth muscle contraction, the citrate cycle (TCA cycle), and other mitochondria-related metabolism signaling (Fig. 3H). Specifically, the TCA cycle emerged as a central hub of mitochondrial energy metabolism, with its gene expression upregulation directly reflecting mitochondrial functional activation. These transcriptional regulatory effects not only demonstrate Set’s direct impact on VSMCs’ phenotypic switching but also imply the potential involvement of ferroptosis and mitochondrial metabolic signaling. To dissect the multilayered regulatory network of Set, we systematically analyzed three critical pathways. Heatmap matrix visualization revealed distinct expression patterns. Set-treated group exhibited significant upregulation of genes associated with vascular smooth muscle contraction and mitopathway, while showing downregulation of core ferroptosis genes, compared to PDGF-BB-stimulated controls (Fig. 3I–K). Collectively, these multi-omics data established that Set exerted pleiotropic regulatory effects through concurrent modulation of vascular contraction, ferroptosis, and mitochondrial metabolism, thereby reconstructing mitochondrial energy metabolic networks to maintain vascular smooth muscle homeostasis.

### Set restrains PDGF-BB-induced functional enhancement and phenotypic switching of VSMCs via inhibiting ferroptosis

**Figure 4.**
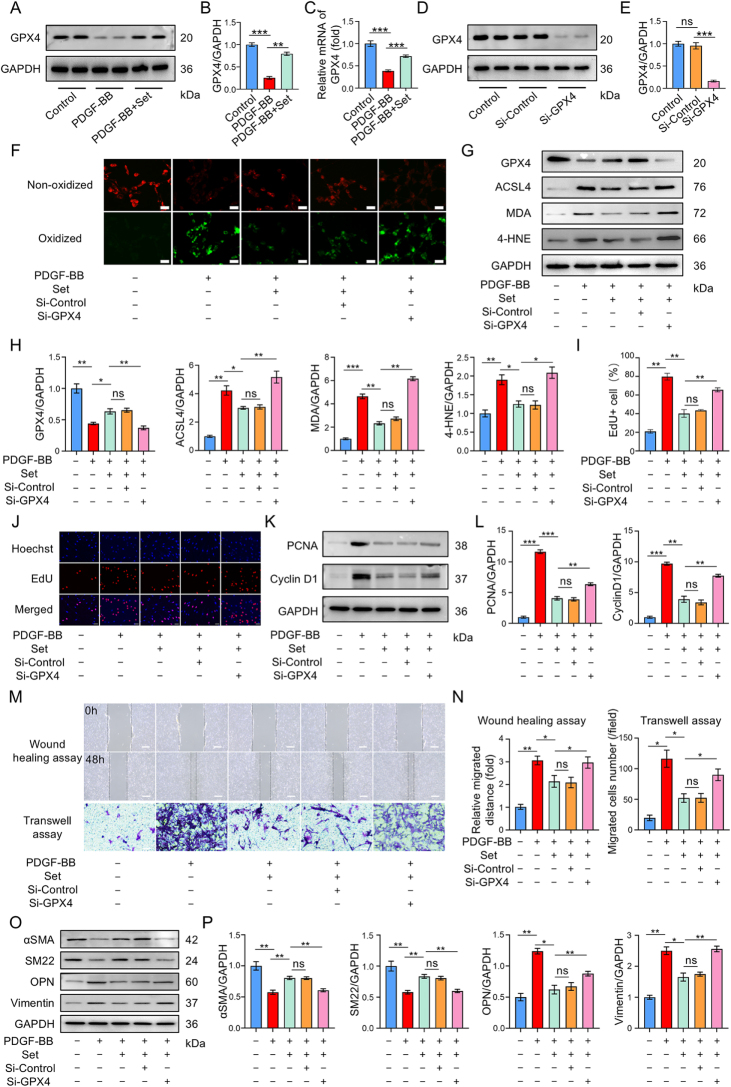
Set suppressed phenotypic transformation of VSMCs by targeting ferroptosis *in vitro*. (A) Representative western blots of GPX4 in VSMCs from the Control, PDGF-BB, and PDGF-BB + Set groups. (B) Quantified protein analysis of GPX4 in VSMCs from the Control, PDGF-BB, and PDGF-BB + Set groups. (C) RT-qPCR analyses of GPX4 in VSMCs from the Control, PDGF-BB, and PDGF-BB + Set groups. (D) Representative western blots of GPX4 in VSMCs from the Control, Si-Control, and Si-GPX4 groups. (E) Quantified protein analysis of GPX4 and GAPDH in the Control, Si-Control, and Si-GPX4 groups. (F) Lipid peroxidation assessment by BODIPY-C11581/591 probe fluorescence. The red signal represented the nonoxidized form of BODIPY-C11581/591 probe, while the green signal reflected the oxidized state of the same probe. Scale bar = 50 μm. (G) Representative western blots of GPX4, ACSL4, MDA, 4-HNE, and GAPDH from each group. (H) Quantified protein analysis of GPX4, ACSL4, MDA, and 4-HNE in each group. (I and J) Representative images of fluorescent EdU assays in VSMCs from each group. Hoechst was used to stain nuclei (blue). EdU dye was incorporated into proliferative cells (red). (K) Representative western blots of PCNA, cyclin D1, and GAPDH from each group. (L) Quantified protein analysis of PCNA, cyclin D1, and GAPDH in each group. (M) Representative images of wound healing assays and Transwell assays in each group. The relative migrated distance at two time points (0 h and 48 h) was measured and analyzed. Scale bar = 200 μm. (N) Representative images of Transwell assays in each group. The number of migrated cells in per field was counted and analyzed. Scale bar = 50 μm. (O) Representative western blots of αSMA, SM22, OPN, vimentin, and GAPDH from each group. (P) Quantified expression of αSMA, SM22, OPN, and vimentin in each group. All biological experiments were repeated three times. The data were presented as the mean ± SD. *P < 0.05, **P < 0.01, ***P < 0.001; ns, not significant.

The previous KEGG analysis indicated that ferroptosis plays a critical role in mediating Set’s effects on VSMCs. Western blot and RT-qPCR analyses confirmed that PDGF-BB-induced downregulation of GPX4 in VSMCs was significantly reversed by Set treatment (Fig. 4A–C). Therefore, we speculated that the inhibitory effect of Set on the proliferation, migration, and phenotypic transformation of VSMCs is exerted through inhibition of ferroptosis. To verify this hypothesis, si-GPX4 and si-Control were introduced into cultured VSMCs. The constructed si-GPX4 successfully inhibited the expression of GPX4 in VSMCs (Fig. 4D and E). Immunofluorescence detection using the BODIPY-C11 581/591 probe showed that Set treatment downregulated the level of lipid peroxidation in dedifferentiated VSMCs (Fig. 4F). The ferroptosis signaling pathway-related molecules were detected by western blot experiments. The results showed that Set treatment upregulated the expression level of GPX4 while it decreased the expression of ACSL4, MDA, and 4-HNE (Fig. 4G and H). However, after knocking down GPX4 by using si-GPX4, the ability of Set to reduce lipid peroxidation level was weakened (Fig. 4G and H). The EdU experiment demonstrated that GPX4 silencing impaired the inhibitory effect of Set on the proliferation of dedifferentiated VSMCs (Fig. 4I and J). Consistently, Set-induced suppression of proliferation markers PCNA and Cyclin D1 was partially reversed following GPX4 depletion (Fig. 4K and L). Similarly, the results of wound healing and Transwell assays also demonstrated that silencing GPX4 partially alleviated the inhibitory effect on the migration ability of dedifferentiated VSMCs (Fig. 4M and N). Phenotypic marker analyses indicated that GPX4 knockdown prevented Set-induced restoration of contractile markers (αSMA, SM22α) and suppressed synthetic phenotypic markers (OPN, vimentin) (Fig. 4O and P).

### Set treatment improves PGC-1α/NRF2/TFAM-mediated mitochondrial biogenesis to target ferroptosis and inhibits dedifferentiated VSMCs

**Figure 5.**
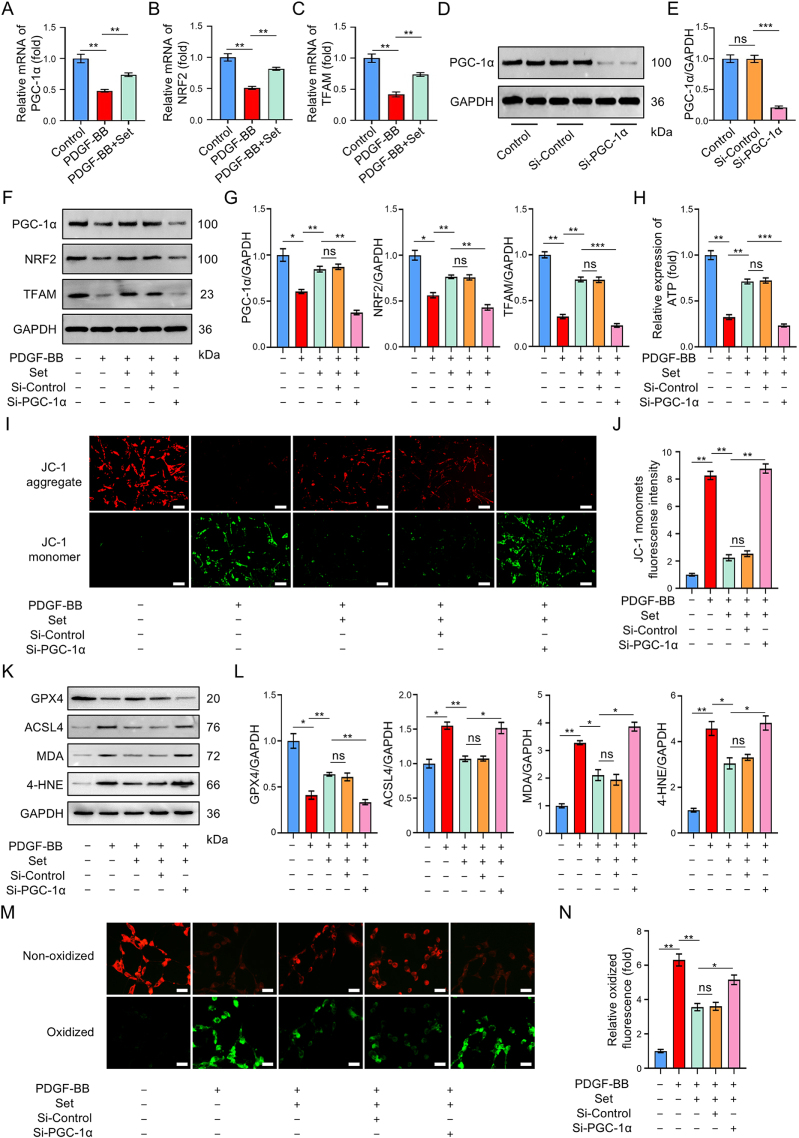
Set treatment activated PGC-1α/NRF2/TFAM pathways to restrain ferroptosis and phenotypic transformation of VSMCs *in vitro*. (A–C) RT-qPCR analyses of PGC-1α, NRF2, and TFAM in VSMCs from the Control, PDGF-BB, and PDGF-BB + Set groups. (D) Representative western blots of PGC-1α and GAPDH from the Control, PDGF-BB, and PDGF-BB + Set groups. (E) Quantified protein analysis of PGC-1α in VSMCs from the Control, PDGF-BB, and PDGF-BB + Set groups. (F) Representative western blots of PGC-1α, NRF2, TFAM, and GAPDH in VSMCs from each group. (G) Quantified protein analysis of PGC-1α, NRF2, and TFAM in each group. (H) Quantified expression of ATP in VSMCs of each group. (I) Mitochondrial membrane potential detection using a JC-1 probe. JC-1 appeared as a polymer in normal mitochondria (red), and as a monomer after mitochondrial membrane potential declined (green). Scale bar = 50 μm. (J) The mean green fluorescence intensity of JC-1 in each group. (K) Representative western blots of GPX4, ACSL4, MDA, 4-HNE, and GAPDH in VSMCs from each group. (L) Quantified protein analysis of GPX4, ACSL4, MDA, and 4-HNE in each group. (M) Lipid peroxidation of VSMCs after PGC-1α knocking down was evaluated by BODIPY-C11581/591 probe fluorescence. The green signal represented the oxidized form of BODIPY-C11581/591 probe. Scale bar = 50 μm. (N) Quantified expression of the mean green fluorescence intensity in each group. All biological experiments were repeated three times. The data were presented as the mean ± SD. * *P* < 0.05, ** *P* < 0.01, *** *P* < 0.001; ns, not significant.

KEGG enrichment analysis of upregulated DEG revealed the activation of mitochondria-related metabolism signaling after Set treatment. To explore the relationship between Set, mitochondria biogenesis, and ferroptosis in AD treatment, we performed correlation heat map analysis from transcriptomic data, and the results showed that the genes of mitopathways were significantly negatively associated with those of ferroptosis (Supplemental Digital Content Figure S3, available at: http://links.lww.com/JS9/F489). It has been reported that selenium supplementation can significantly activate PGC-1α expression. PGC-1α can induce the expression of *NRF2* and *TFAM* genes and jointly regulate mitochondrial biogenesis. The RT-qPCR experiment confirmed that the mRNA levels of PGC-1α, NRF2, and TFAM in dedifferentiated VSMCs were significantly downregulated, and their expression levels could be restored by Set treatment (Fig. 5A–C). Therefore, we speculated that the inhibitory effect of Set on ferroptosis is exerted through PGC-1α/NRF2 pathway. To verify this hypothesis, si-PGC-1α was introduced into the cultured VSMCs. Western blot analysis confirmed that the constructed si-PGC-1α successfully inhibits the expression of PGC-1α in VSMCs (Fig. 5D and E), and PGC-1α silencing restrains Set-induced upregulated expressions of PGC-1α, NRF2, and TFAM in dedifferentiated VSMCs (Fig. 5F and G). ATP content detection demonstrated that Set treatment could enhance ATP synthesis in mitochondria, while it was suppressed by PGC-1α silencing (Fig. 5H). The mitochondrial membrane potential level was detected by using the JC-1 fluorescent probe, and the results suggested that Set treatment could increase the mitochondrial membrane potential level, but likewise, PGC-1α silencing reversed that effect (Fig. 5I and J). Therefore, inhibition of PGC-1α expression could impair the ability of Set to activate PGC-1α/NRF2/TFAM pathways and induce mitochondrial biogenesis. Then, ferroptosis-related molecules were detected after PGC-1α inhibition using western blot experiments, and the results indicated that the decreased ferroptosis stress in dedifferentiated VSMCs caused by Set was rescued (Fig. 5K and L). Besides, PGC-1α silencing also restored the lipid peroxidation level of dedifferentiated VSMCs (Fig. 5M and N). These results demonstrated that the impairment of mitochondrial homeostasis can activate ferroptosis and disrupt the inhibitory effect of Set on dedifferentiated VSMCs.

### Set treatment activates the PGC-1α/NRF2/TFAM pathway and inhibits the ferroptosis signal in AD models

**Figure 6.**
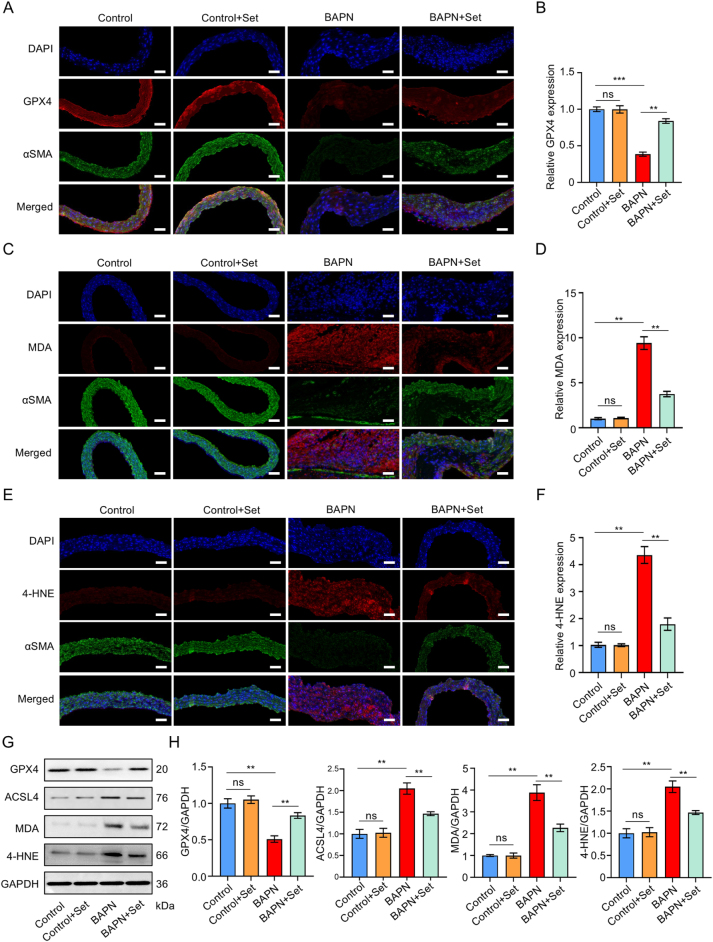
Set treatment inhibited the ferroptosis signal *in vivo* experiments. (A) GPX4 expression detection by immunofluorescence staining of paraffin sections of aortic tissue from the Control, Control + Set, BAPN, and BAPN + Set groups. (B) Quantified analysis of the mean red fluorescence intensity in the Control, Control + Set, BAPN, and BAPN + Set groups (*n* =5 per group). (C) MDA expression detection by immunofluorescence staining of paraffin sections of aortic tissue from the Control, Control + Set, BAPN, and BAPN + Set groups. (D) Quantified analysis of the mean red fluorescence intensity in the Control, Control + Set, BAPN, and BAPN + Set groups (*n* = 5 per group). (E) 4-HNE expression detection by immunofluorescence staining of paraffin sections of aortic tissue from the Control, Control + Set, BAPN, and BAPN + Set groups. (F) Quantified analysis of the mean fluorescence intensity of 4-HNE in the Control, Control + Set, BAPN, and BAPN + Set groups (*n* = 5 per group). (G) Representative western blots of GPX4, ACSL4, MDA, 4-HNE, and GAPDH in aortic tissue from the Control, Control + Set, BAPN, and BAPN + Set groups. (**H)** Quantified protein analysis of GPX4, ACSL4, MDA, and 4-HNE in aortic tissue from the Control, Control + Set, BAPN, and BAPN + Set groups (*n* = 3 per group). The data were presented as the mean ± SD. *P < 0.05, **P < 0.01, ***P < 0.001; ns, not significant.

**Figure 7.**
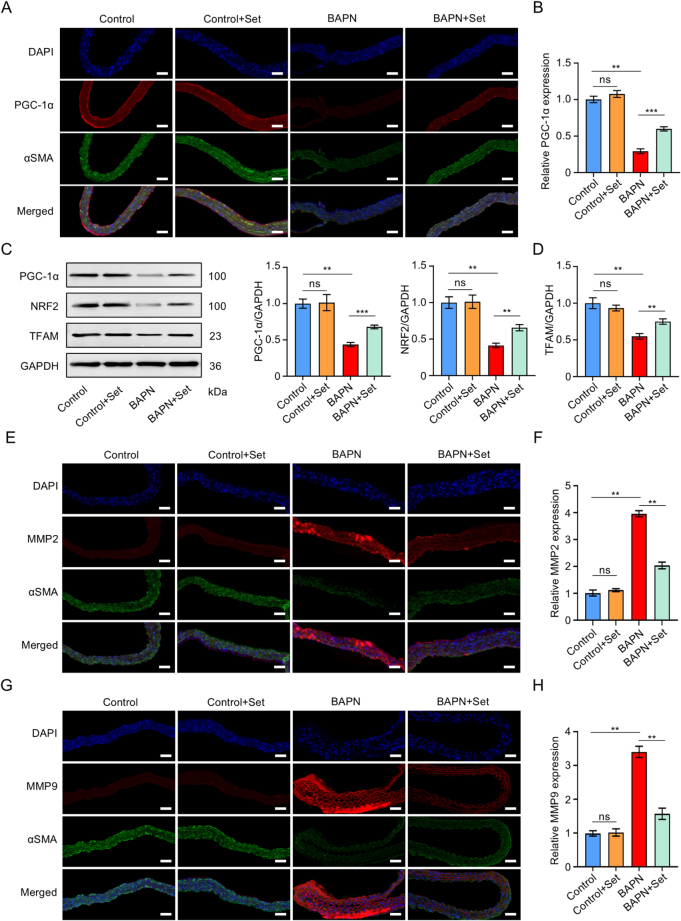
Set treatment activated the PGC-1α/NRF2/TFAM pathway and suppressed migration of VSMCs *in vivo* experiments. (A) PGC-1α expression detection by immunofluorescence staining of paraffin sections of aortic tissues from the Control, Control + Set, BAPN, and BAPN + Set groups. (B) Quantified expression of the mean fluorescence intensity of PGC-1α in aortic tissue from Control, Control + Set, BAPN, and BAPN + Set groups (*n* = 5 per group). (C) Representative western blots of PGC-1α, NRF2, TFAM, and GAPDH from the Control, Control + Set, BAPN, and BAPN + Set groups. (D) Quantified expression of PGC-1α, NRF2, and TFAM in aortic tissue from the Control, Control + Set, BAPN, and BAPN + Set groups (*n* = 3 per group). (E) MMP2 expression detection by immunofluorescence staining of paraffin sections of aortic tissues from the Control, Control + Set, BAPN, and BAPN + Set groups. (F) Quantified expression of the mean fluorescence intensity of MMP2 in the Control, Control + Set, BAPN, and BAPN + Set groups (*n* = 5 per group). (G) MMP9 expression detection by immunofluorescence staining of paraffin sections of aortic tissue from the Control, Control + Set, BAPN, and BAPN + Set groups. (H) Quantified expression of the mean fluorescence intensity of MMP9 in the Control, Control + Set, BAPN, and BAPN + Set groups (*n* = 5 per group). The data were presented as the mean ± SD. *P < 0.05, **P < 0.01, ***P < 0.001; ns, not significant.

The *in vitro* studies have revealed the relationship between PGC-1α/NRF2/TFAM pathway and ferroptosis in Set-inhibited dedifferentiated VSMCs. Whether they still share such expression patterns *in vivo* models requires further verification. Aortic tissue sections were collected for analysis. The results of immunofluorescence showed that, compared to BAPN-treated groups, Set administration significantly upregulated GPX4 expression (Fig. 6A and B), while concurrently suppressing lipid peroxidation markers, including MDA (Fig. 6C and D) and 4-HNE (Fig. 6E and F). These findings were corroborated by western blot quantification showing elevated GPX4 protein levels and reduced ferroptosis stress markers in Set-treated groups (Fig. 6G), and the quantitative analysis also supported these results (Fig. 6H). Furthermore, Set treatment activated mitochondrial biogenesis through the PGC-1α/NRF2 pathway *in vivo*, as evidenced by immunofluorescence staining showing enhanced PGC-1α expression in aortic tissue sections (Fig. 7A and B). The results of western blot analysis suggested significant upregulation of both PGC-1α and its downstream effectors NRF2 and TFAM in the Set-treated groups, compared to BAPN controls (Fig. 7C and D). Notably, as migration markers, matrix metalloproteinases also play a critical role in extracellular matrix degradation and the pathogenesis of AD. Immunofluorescence staining results showed Set administration exhibited inhibitory effects on the expression of matrix metalloproteinase, including both MMP2 (Fig. 7E and F) and MMP9 (Fig. 7G and H).

## Discussion

**Figure 8.**
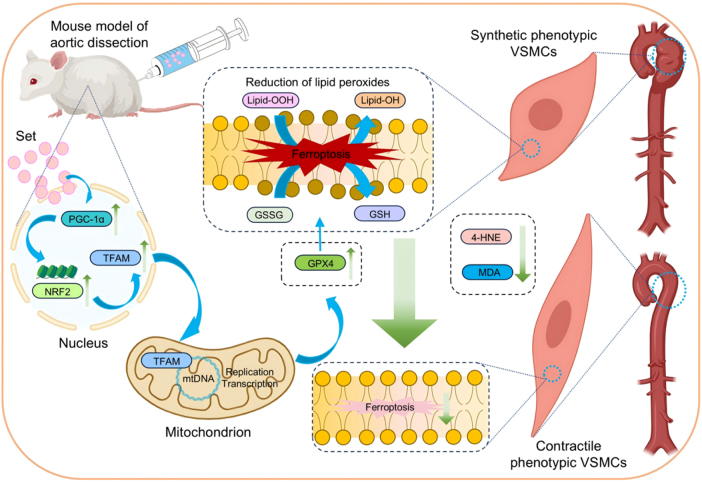
Mechanistic overview of the theraputic effect of Set on AD by activating PGC-1α/NRF2/TFAM axis and mitochondrial biosynthesis, inhibiting ferroptosis, and reversing phenotypic transformation of VSMCs.

AD is a cardiovascular disease that poses a serious threat to life. Once the aorta ruptures, the patient’s survival probability declines precipitously. Current treatment methods mainly rely on surgeries, including open surgeries and endovascular repair. However, these surgeries are highly invasive and carry high risks, and some patients are unable to tolerate them due to reasons such as age and comorbidity. Moreover, there are still some patients who, although having already been diagnosed with AD, do not meet the surgical indications. Existing drug treatments mainly focus on controlling risk factors such as hypertension and hyperlipidemia, and there is a lack of specific drugs targeting AD. Consequently, there is an urgent need to explore novel therapeutic targets and drugs for AD. In this study, the protective effect and potential mechanism of Set on AD were investigated (Fig. 8). The principal findings of this study are presented as follows: (1) Set treatment attenuated the progression of AD *in vivo*. It notably reduces both the incidence and mortality rates of AD and suppresses the aortic dilation; (2) Set reversed phenotypic switching of VSMCs by upregulating the expression of GPX4 and further inhibiting ferroptosis; (3) Set enhanced the transcriptional activity of GPX4 through the activation of the PGC-1α/NRF2 pathway-mediated mitochondrial biogenesis.

Set is a significant organic selenium compound. In recent years, its biological functions and clinical applications have garnered substantial interest. Accumulating evidence indicated that Set exerts notable antioxidant, anti-inflammatory, antiaging, and anti-apoptotic effects, while also inhibiting ferroptosis^[27,31–33]^. A relevant study demonstrated that Set can relieve the oxidative stress induced by deoxynivalenol (DON) by activating the Keap1/NRF2 signaling level in the small intestinal epithelium^[31]^. In addition, another study has indicated that Set protects against Escherichia coli-induced endometritis by activating the PPAR-γ/NF-κB pathway to suppress inflammation and necroptosis^[32]^. Moreover, Set has been shown to alleviate PM2.5-induced cellular senescence in the lung by attenuating inflammatory responses mediated by the cGAS/STING/NF-κB pathway^[33]^. In this study, we demonstrate that Set effectively mitigated the AD progression. However, in the current study, the BAPN-induced mice were merely treated with Set at a dosage of 200 µg/kg, which constitutes a notable limitation. In the subsequent investigations, we will carry out research using drugs at a wider range of concentrations.

The phenotypic transformation of VSMCs plays a critical role in AD progression. Synthetic VSMCs overproduce extracellular matrix such as collagen, leading to vascular stiffening, wall thickening, and loss of elasticity. Meanwhile, they secrete a variety of inflammatory factors that exacerbate the inflammatory microenvironment, creating vascular wall inflammation, accelerating the pathological process of the blood vessel wall, and promoting the continuous AD progression. Previous studies have reported that inhibiting the phenotypic transformation of normal VSMCs represents a crucial therapeutic strategy for AD^[11,34,35]^. Studies indicate that the activation of hypoxia-inducible factor (HIF) promotes the occurrence and development of AD by inducing the phenotypic transformation of VSMCs. PX478 attenuates the progression of AD by inhibiting HIF^[9]^. Moreover, another study reported that aldehyde dehydrogenase 2 (ALDH2) can increase the level of microRNA-31-5p (miR-31-5p), which accelerates the progression of AD by triggering the phenotypic transformation of VSMCs^[36]^. In addition, a previous study has found that the phenotypic transformation of VSMCs mediated by the upregulation of serum/glucocorticoid-regulated kinase 1 (SGK1) promotes the progression of AD^[11]^. Furthermore, microRNA-145 (miR-145) inhibited AD by weakening the phenotypic transformation of VMSCs. In this study, we demonstrate that Set inhibited the progression of AD by suppressing the proliferation, migration, and phenotypic switching of VSMCs *in vivo* and *in vitro* experiments.

Ferroptosis, a newly identified form of programmed cell death, plays a critical role in the development of AD^[20,22,37,38]^. Studies demonstrated that miR-1909-5p induces ferroptosis by downregulating GPX4, thus accelerating AD progression^[21]^. Oxidative stress-induced damage to normal VSMCs through ferroptosis mediated by HIF-1α/heme oxygenase-1, thereby aggravating AD^[39]^. Furthermore, BRD4770, a novel ferroptosis inhibitor, can restrain the progression of AD through the downregulation of ferroptosis signals^[38]^. Moreover, a previous study has found that exosomes programmed and released by 3D-printed hydrogels inhibited AD by suppressing ferroptosis of VSMCs^[37]^. In addition, upregulation of lactate dehydrogenase A can also inhibit the progression of AD by suppressing the ferroptosis signaling pathway^[40]^. All these findings suggested that downregulating ferroptosis signals may be a potential target for the treatment of AD. Currently, numerous ferroptosis inhibitors have been discovered. Ferrostatin-1 was a relatively recognized ferroptosis inhibitor, yet its mechanism of action and biological safety remained unclear^[18,41–45]^.

The regulatory effect of Set on ferroptosis has been reported in many studies at present. For example, Set attenuates ferroptosis in T-2 toxin-exposed articular chondrocytes and inhibits ferroptosis of small intestinal epithelial cells caused by ammonia exposure^[46,47]^. In this study, we observed that Set inhibited the ferroptosis signals in VSMCs induced by PDGF-BB. Furthermore, Set upregulates the expression level of GPX4 in VSMCs, while reducing the levels of lipid peroxidation products, namely MDA and 4-HNE, simultaneously. Multiple studies have reported that Set can upregulate the expression level of GPX4 to inhibit ferroptosis signals^[26,27,46]^. Consequently, we propose a hypothesis that the inhibitory effect of Set on dedifferentiated VSMCs was achieved through the upregulation of GPX4. In order to verify this hypothesis, si-GPX4 was transfected into the dedifferentiated VSMCs. The results of the reversal experiment showed that the inhibitory effect of Set on ferroptosis and the reversing effect on the phenotypic transformation of dedifferentiated VSMCs were weakened. However, considering that only 50 μM of Set was used in the cell experiments conducted in this study, the effectiveness and specificity of Set in inhibiting ferroptosis signals still need to be clarified through further research. Subsequently, different concentrations of Set should be used to evaluate its protective effects. Additionally, Set has been utilized in a number of clinical trials (NCT00736645, NCT01497431). The experimental data derived from these trials have served to verify the drug safety of Set. In conclusion, our research data supported that ferroptosis may be a potential target for the prevention and treatment of AD. Moreover, we have also ascertained that Set was an effective and safe drug capable of inhibiting AD. It achieved this by activating GPX4, downregulating ferroptosis signals, and suppressing the phenotypic transformation of dedifferentiated VSMCs.

In recent years, given the potential protective effects of PGC-1α activation on the cardiovascular system, the PGC-1α/NRF2 signaling pathway has gained widespread attention. According to relevant literature reports, the ameliorative effects of melatonin and resveratrol on diabetic cardiomyopathy are achieved through the PGC-1α/NRF2 signaling pathway^[48,49]^. Additionally, PGC-1α activation improved the myocardial infarction model of male Sprague Dawley rats^[50,51]^. It has been pointed out that the overexpression of high mobility group box 2 (HMGB2) aggravated the progression of AD by inhibiting PGC-1α expression^[52]^. In contrast, the inhibition of HMGB2 protected against AD progression. Besides, it has also been reported in studies that downregulating the PGC-1α signal level involved in mitochondrial biogenesis exacerbated the ferroptosis signal of lung epithelial cells^[53]^. Moreover, studies have confirmed that the improvement effect of Set treatment on the mitochondrial biogenesis of placental trophoblast cells is achieved by upregulating the expressions of PGC-1α and NRF2^[28]^. In addition, selenium supplementation treatment can improve diabetic myocardial hypertrophy by upregulating the level of PGC-1α^[54]^. In this study, we found that Set upregulated the levels of PGC-1α/NRF2/TFAM in PDGF-BB-induced VSMCs, improved the mitochondrial biogenesis level and ATP content of the cells, and simultaneously inhibited the ferroptosis signal. Based on the preliminary research, we speculate that the ferroptosis inhibition of dedifferentiated VSMCs by Set was exerted through the upregulation of PGC-1α. To confirm this hypothesis, si-PGC-1α was transfected into the dedifferentiated VSMCs. The results of the rescue experiment showed that the inhibitory effect of Set on ferroptosis was eliminated. In conclusion, our research data supported that PGC-1α may be a potential target for the prevention and treatment of AD. Moreover, we have also confirmed that the inhibitory effect of Set on ferroptosis signals by upregulating the expression of GPX4 is achieved through upregulating the expression levels of PGC-1α/NRF2.

Thoracic AD is mainly divided into type A and type B. Type A AD, involving the ascending aorta (close to the heart), requires emergency surgery. Type B AD, limited to the descending aorta (far from the heart), can be treated with medication or interventional therapy. Our findings demonstrate that Set significantly reduces both incidence and mortality of BAPN-induced AD. Therefore, the positive significance of Set lies in providing a new option for the conservative treatment of type B AD, especially for patients with AD who are asymptomatic but already have imaging changes, or those at risk of developing AD. Therefore, Set may prevent the acute occurrence of AD or improve the symptoms of the chronic phase by reversing the pathological changes of VSMCs.

There are some limitations in our research. Firstly, due to the limitations of clinical data, we are currently unable to obtain the correlation of human body data (such as the Set plasma level of patients and dietary selenium intake). In the next step, we will further carry out related work. Secondly, the knockdown experiments of GPX4 and PGC-1α were only conducted *in vitro* and not *in vivo*. In the future, we will conduct more concentrated drug experiments and explorations.

In conclusion, Set inhibits the ferroptosis stress by activating the PGC-1α/NRF2 signal, thereby upregulating GPX4 expression. This mechanism suppresses the function and phenotypic transformation of VSMCs, ultimately inhibiting the progression of AD. Our current research has successfully identified a potential therapeutic drug for AD and has comprehensively elucidated the specific mechanism, which is of great significance to AD treatment.

## Data Availability

Data will be made available on request.

## References

[1] LeMaire SA Russell L. Epidemiology of thoracic aortic dissection. Nat Rev Cardiol 2011;8:103–13. 21173794 10.1038/nrcardio.2010.187

[2] Pape LA Awais M Woznicki EM. Presentation, diagnosis, and outcomes of acute aortic dissection: 17-year trends from the international registry of acute aortic dissection. J Am Coll Cardiol 2015;66:350–58. 26205591 10.1016/j.jacc.2015.05.029

[3] Harris KM Nienaber CA Peterson MD. Early mortality in type a acute aortic dissection: insights from the international registry of acute aortic dissection. JAMA Cardiol 2022;7:1009–15. 36001309 10.1001/jamacardio.2022.2718 PMC9403853

[4] Xia L Sun C Zhu H. Melatonin protects against thoracic aortic aneurysm and dissection through SIRT1-dependent regulation of oxidative stress and vascular smooth muscle cell loss. J Pineal Res 2020;69:e12661. 32329099 10.1111/jpi.12661

[5] Jiang H Zhao Y Jin M. Colchicine inhibits smooth muscle cell phenotypic switch and aortic dissection in mice-brief report. Arterioscler Thromb Vasc Biol 2025;45:979–84. 40177774 10.1161/ATVBAHA.124.322252

[6] Zhao K Zhu H He X. Senkyunolide I ameliorates thoracic aortic aneurysm and dissection in mice via inhibiting the oxidative stress and apoptosis of endothelial cells. Biochim Biophys Acta Mol Basis Dis 2023;1869:166819. 37499930 10.1016/j.bbadis.2023.166819

[7] Guo Y Che Y Zhang X. Cannabidiol protects against acute aortic dissection by inhibiting macrophage infiltration and PMAIP1-induced vascular smooth muscle cell apoptosis. J Mol Cell Cardiol 2024;189:38–51. 38387723 10.1016/j.yjmcc.2024.02.006

[8] Zhou C Lin Z Cao H. Anxa1 in smooth muscle cells protects against acute aortic dissection. Cardiovasc Res 2022;118:1564–82. 10.1093/cvr/cvab109 33757117

[9] Chen Y Li X Wang Z. Iron deficiency affects oxygen transport and activates HIF1 signaling pathway to regulate phenotypic transformation of VSMC in aortic dissection. Mol Med 2024;30:90. 38886644 10.1186/s10020-024-00859-y PMC11184844

[10] Qiu ZH He J Chai TC. miR-145 attenuates phenotypic transformation of aortic vascular smooth muscle cells to prevent aortic dissection. J Clin Lab Anal 2021;35:e23773. 34767671 10.1002/jcla.23773 PMC8649326

[11] Leng S Li H Zhang P. SGK1-mediated vascular smooth muscle cell phenotypic transformation promotes thoracic aortic dissection progression. Arterioscler Thromb Vasc Biol 2025;45:238–59. 39633576 10.1161/ATVBAHA.124.321421 PMC11748913

[12] Bai Y Luo Y Yuan Y. Ferroptosis: a novel therapeutic warrior in the battle against leukemia. Apoptosis 2025;30:1776–95. 40488837 10.1007/s10495-025-02130-z

[13] Long H Zhu W Wei L Zhao J. Iron homeostasis imbalance and ferroptosis in brain diseases. MedComm (2020) 2023;4:e298. 37377861 10.1002/mco2.298 PMC10292684

[14] Yang M Luo H Yi X Wei X Jiang DS. The epigenetic regulatory mechanisms of ferroptosis and its implications for biological processes and diseases. MedComm (2020) 2023;4:e267. 37229485 10.1002/mco2.267 PMC10203370

[15] Chen Y Yi X Wei X Jiang DS. Ferroptosis: a novel pathological mechanism of aortic dissection. Pharmacol Res 2022;182:106351. 35835368 10.1016/j.phrs.2022.106351

[16] Zhu L Liu Z Liu J. NCOA4 linked to endothelial cell ferritinophagy and ferroptosis:a key regulator aggravate aortic endothelial inflammation and atherosclerosis. Redox Biol 2025;79:103465. 39700692 10.1016/j.redox.2024.103465 PMC11729014

[17] Liang D Feng Y Zandkarimi F. Ferroptosis surveillance independent of GPX4 and differentially regulated by sex hormones. Cell 2023;186:2748–64.e22. 10.1016/j.cell.2023.05.003 PMC10330611 37267948

[18] Fang X Ardehali H Min J Wang F. The molecular and metabolic landscape of iron and ferroptosis in cardiovascular disease. Nat Rev Cardiol 2023;20:7–23. 35788564 10.1038/s41569-022-00735-4 PMC9252571

[19] Zhang Y Xin L Xiang M. The molecular mechanisms of ferroptosis and its role in cardiovascular disease. Biomed Pharmacother 2022;145:112423. 10.1016/j.biopha.2021.112423 34800783

[20] Li N Yi X He Y. Targeting ferroptosis as a novel approach to alleviate aortic dissection. Int J Biol Sci 2022;18:4118–34. 35844806 10.7150/ijbs.72528 PMC9274489

[21] Tao Y Li G Wang Z. MiR-1909-5p targeting GPX4 affects the progression of aortic dissection by modulating nicotine-induced ferroptosis. Food Chem Toxicol 2024;191:114826. 10.1016/j.fct.2024.114826 38897284

[22] Qi Z Wang QG Huang MX. Dual functions of silibinin in attenuating aortic dissection via regulating iron homeostasis and endoplasmic reticulum stress against ferroptosis. Cell Death Dis 2024;15:900. 39695164 10.1038/s41419-024-07309-x PMC11655547

[23] He Y Wang X Chen S. SP2509 functions as a novel ferroptosis inhibitor by reducing intracellular iron level in vascular smooth muscle cells. Free Radic Biol Med 2024;219:49–63. 38608823 10.1016/j.freeradbiomed.2024.04.220

[24] Ge MM Li DY Wang L. Naringenin promoted spinal microglia M2 polarization in rat model of cancer-induced bone pain via regulating AMPK/PGC-1α signaling axis. Biomed Pharmacother 2022;149:112912. 10.1016/j.biopha.2022.112912 35856853

[25] Duan C Li B Liu H. Sirtuin1 suppresses calcium oxalate nephropathy via inhibition of renal proximal tubular cell ferroptosis through PGC-1α-mediated transcriptional coactivation. Adv Sci (Weinh) 2024;11:e2408945. 10.1002/advs.202408945 PMC11672264 39498889

[26] Dong B Jiang Y Shi B Zhang Z, and Zhang Z. Selenomethionine alleviates decabromodiphenyl ether-induced oxidative stress and ferroptosis via the NRF2/GPX4 pathway in the chicken brain. J Hazard Mater 2024;465:133307. 10.1016/j.jhazmat.2023.133307 38154185

[27] Sun J Xie X Song Y. Selenomethionine in gelatin methacryloyl hydrogels: modulating ferroptosis to attenuate skin aging. Bioact Mater 2024;35:495–516. 38404642 10.1016/j.bioactmat.2024.02.013 PMC10885793

[28] Khera A Dong LF Holland O. Selenium supplementation induces mitochondrial biogenesis in trophoblasts. Placenta 2015;36:863–69. 26154583 10.1016/j.placenta.2015.06.010

[29] Agha RA Mathew G Rashid R. Transparency in the reporting of Artificial Intelligence – the TITAN guideline. Prem J Sci 2025;10:100082.

[30] Kilkenny C Browne WJ Cuthill IC Emerson M Altman DG. Improving bioscience research reporting: the ARRIVE guidelines for reporting animal research. PLoS Biol. 2010;8:e1000412. 20613859 10.1371/journal.pbio.1000412 PMC2893951

[31] Zhu C Liang S Zan G. Selenomethionine alleviates DON-induced oxidative stress via modulating keap1/Nrf2 signaling in the small intestinal epithelium. J Agric Food Chem 2023;71:895–904. 36535023 10.1021/acs.jafc.2c07885

[32] Cao L Gao S Liu J Wang J, and Qin R. Selenomethionine protects against Escherichia coli-induced endometritis by inhibiting inflammation and necroptosis via regulating the PPAR-γ/NF-κB pathway. Chem Biol Interact 2023;379:110532. 10.1016/j.cbi.2023.110532 37150495

[33] Wang X Lu W Xia X. Selenomethionine mitigate PM2.5-induced cellular senescence in the lung via attenuating inflammatory response mediated by cGAS/STING/NF-κB pathway. Ecotoxicol Environ Saf 2022;247:114266. 10.1016/j.ecoenv.2022.114266 36334339

[34] Dang Z Li H Xue S. Histone deacetylase 9-mediated phenotypic transformation of vascular smooth muscle cells is a potential target for treating aortic aneurysm/dissection. Biomed Pharmacother 2024;173:116396. 10.1016/j.biopha.2024.116396 38460370

[35] Li M Li G Yang Y. piRNA-823 is a novel potential therapeutic target in aortic dissection. Pharmacol Res 2023;196:106932. 37739144 10.1016/j.phrs.2023.106932

[36] Yang K Ren J Li X. Prevention of aortic dissection and aneurysm via an ALDH2-mediated switch in vascular smooth muscle cell phenotype. Eur Heart J 2020;41:2442–53. 32428930 10.1093/eurheartj/ehaa352

[37] Wang W Liu Q Yang Q. 3D-printing hydrogel programmed released exosomes to restore aortic medial degeneration through inhibiting VSMC ferroptosis in aortic dissection. J Nanobiotechnol 2024;22:600. 10.1186/s12951-024-02821-w PMC11453022 39367412

[38] Li H Wang PF Luo W. CD36-mediated ferroptosis destabilizes CD4+ T cell homeostasis in acute stanford type-A aortic dissection. Cell Death Dis 2024;15:669. 39266539 10.1038/s41419-024-07022-9 PMC11392947

[39] Song W Chen Y Qin L. Oxidative stress drives vascular smooth muscle cell damage in acute stanford type Aaortic dissection through HIF-1α/HO-1 mediated ferroptosis. Heliyon 2023;9:e22857. 38125409 10.1016/j.heliyon.2023.e22857 PMC10730757

[40] Feng X Yi X Huo B. Lactate dehydrogenase A is a novel positive regulator of vascular smooth muscle cell ferroptosis during aortic dissection. Antioxid Redox Signal 2025;42:378–92. 39605213 10.1089/ars.2024.0585

[41] Tong J Lan XT Zhang Z. Ferroptosis inhibitor liproxstatin-1 alleviates metabolic dysfunction-associated fatty liver disease in mice: potential involvement of PANoptosis. Acta Pharmacol Sin 2023;44:1014–28. 36323829 10.1038/s41401-022-01010-5 PMC10104837

[42] Ryan F Blex C Ngo TD. Ferroptosis inhibitor improves outcome after early and delayed treatment in mild spinal cord injury. Acta Neuropathol 2024;147:106. 38907771 10.1007/s00401-024-02758-2 PMC11193702

[43] Huang Q Ru Y Luo Y. Identification of a targeted ACSL4 inhibitor to treat ferroptosis-related diseases. Sci Adv 2024;10:eadk1200. 38552012 10.1126/sciadv.adk1200 PMC10980261

[44] Liu P Feng Y Li H. Ferrostatin-1 alleviates lipopolysaccharide-induced acute lung injury via inhibiting ferroptosis. Cell Mol Biol Lett 2020;25:10. 32161620 10.1186/s11658-020-00205-0 PMC7045739

[45] Zhou Q Yang L Li T. Mechanisms and inhibitors of ferroptosis in psoriasis. Front Mol Biosci 2022;9:1019447. 36188212 10.3389/fmolb.2022.1019447 PMC9520612

[46] Yu FF Zuo J Wang M. Selenomethionine alleviates T-2 toxin-induced articular chondrocyte ferroptosis via the system Xc-/GSH/GPX4 axis. Ecotoxicol Environ Saf 2025;290:117569. 39700767 10.1016/j.ecoenv.2024.117569

[47] Zhang X Gu L Chen Y Wang T Xing H. L-selenomethionine inhibits small intestinal ferroptosis caused by ammonia exposure through regulating ROS-mediated iron metabolism. Ecotoxicol Environ Saf 2025;289:117477. 39657382 10.1016/j.ecoenv.2024.117477

[48] Ding M Feng N Tang D. Melatonin prevents Drp1-mediated mitochondrial fission in diabetic hearts through SIRT1-PGC1α pathway. J Pineal Res 2018;65:e12491. 10.1111/jpi.12491 PMC6099285 29575122

[49] Fang WJ Wang CJ He Y Zhou YL Peng XD Liu SK. Resveratrol alleviates diabetic cardiomyopathy in rats by improving mitochondrial function through PGC-1α deacetylation. Acta Pharmacol Sin 2018;39:59–73. 28770830 10.1038/aps.2017.50 PMC5758665

[50] Gormaz JG Quintremil S Rodrigo R. Cardiovascular disease: a target for the pharmacological effects of quercetin. Curr Top Med Chem 2015;15:1735–42. 25915608 10.2174/1568026615666150427124357

[51] Han X Yang Y Zhang M. Protective effects of 6-gingerol on cardiotoxicity induced by arsenic trioxide through AMPK/SIRT1/PGC-1α signaling pathway. Front Pharmacol 2022;13:868393. 35571130 10.3389/fphar.2022.868393 PMC9096219

[52] Zheng Y Yao M Chen S. HMGB2 promotes smooth muscle cell proliferation through PPAR-γ/PGC-1α pathway-mediated glucose changes in aortic dissection. Atherosclerosis 2024;399:119044. 10.1016/j.atherosclerosis.2024.119044 39531897

[53] Zhu L Zhu D Ran J. Autophagy aggravates multi-walled carbon nanotube-induced ferroptosis by suppressing PGC-1 dependent-mitochondrial biogenesis in lung epithelial cells. Chem Biol Interact 2024;400:111158. 10.1016/j.cbi.2024.111158 39033796

[54] Mushtaq I Bashir Z Sarwar M. N-acetyl cysteine, selenium, and ascorbic acid rescue diabetic cardiac hypertrophy via mitochondrial-associated redox regulators. Molecules 2021;26:7285. 34885867 10.3390/molecules26237285 PMC8659237

